# Antibacterial coating of orthodontic elastomeric ligatures with silver and bismuth nanofilms by magnetron sputtering: A feasibility study

**DOI:** 10.1002/cre2.864

**Published:** 2024-03-03

**Authors:** Andrea Schubert, Carolin Griesmüller, Nikolaus Gersdorff, Ralf Bürgers, Bernhard Wiechens, Torsten Wassmann

**Affiliations:** ^1^ Department of Prosthodontics University Medical Center Goettingen Goettingen Germany; ^2^ Department of Orthodontics University Medical Center Goettingen Goettingen Germany

**Keywords:** elastomeric ligatures, magnetron sputtering, microbial adhesion, nanoparticles

## Abstract

**Objectives:**

Magnetron sputtering was evaluated to equip surfaces of orthodontic elastomeric ligatures with silver and bismuth nanofilms.

**Material and Methods:**

Antibacterial properties were evaluated by the adhesion of *Streptococcus mutans*. Polyurethane‐based elastomeric ligatures were coated with silver and bismuth nanofilms via direct current magnetron sputtering. Surface roughness (*R*_a_) and surface‐free energy (SFE) were assessed. Coated specimens were incubated with *S. mutans* for 2 h. Adhering bacteria were visualized by Hoechst staining and quantified by an ATP‐based luminescence assay. One‐way analysis of variance with Tukey post hoc testing and Pearson correlation analysis were performed (*p* < .05) to relate bacterial adhesion to surface roughness and surface‐free energy.

**Results:**

Elastomeric ligatures were successfully coated with silver and bismuth nanofilms. *R*_a_ was significantly reduced by silver coating. Silver and bismuth coatings showed significantly higher SFE than controls. Adhesion of *S. mutans* was significantly decreased by silver coating. No correlation between bacterial adhesion and SFE was found. Correlation between bacterial adhesion and *R*_a_ was positive but not statistically significant.

**Conclusions:**

Magnetron sputtering proved to be a feasible method to equip orthodontic elastomeric ligatures with silver and bismuth nanofilms. Silver coatings of elastomeric ligatures may reduce white spots and carious lesions in orthodontic patients. Future research is required to stabilize coatings.

## 1 INTRODUCTION

During orthodontic treatment, fixed appliances complicate oral hygiene while simultaneously creating retentive niches for microbial accumulation (Atack et al., 1996; Balenseifen & Madonia, 1970; Gwinnett & Ceen, 1979). While microbial adhesion to dental hard tissue does not necessarily have pathological consequences per se, a dental biofilm can develop cariogenic properties due to a dysbiosis of the oral microbiome (Johansson et al., 2016). An oral dysbiosis can be modulated by dietary habits and is manifested in an altered, potentially pathological composition of dental biofilms (Kahharova et al., 2023; Pitts et al., 2021; Zheng et al., 2021). Among the multiple microorganisms present in dental biofilms, *Streptococcus mutans* is considered a primary causative agent for enamel decalcification due to its ability to produce lactic acid by processing low molecular weight oligosaccharides (Hamada & Slade, 1980; Legéňová & Bujdáková, 2015; Loesche, 1986). In the presence of fixed orthodontic appliances, levels of *S. mutans* are elevated in the saliva and dental plaque of patients (Lundstrom & Krasse, 1987; Pellegrini et al., 2009; Rosenbloom & Tinanoff, 1991). White spots and carious lesions of the adjacent enamel are prevalent adverse side effects of fixed orthodontic treatment (Enaia et al., 2011; Gorelick et al., 1982; Ogaard et al., 1988; Richter et al., 2011; Sundararaj et al., 2015; Tufekci et al., 2011).

Common strategies to prevent enamel decalcification during orthodontic treatment include mechanical plaque debridement, individual hygiene instructions, fluoride application, and the use of antimicrobial mouthwashes (Geiger et al. 1988, 1992; Harvey & Powell, 1981; Srivastava et al., 2013). However, all of these approaches depend on the patient's individual motivation and compliance (Geiger et al. 1988, 1992; Hadler‐Olsen et al., 2012). Orthodontic appliances with antibacterial properties would be of great clinical value to limit bacterial adhesion regardless of patient compliance.

Besides brackets that are bonded to the tooth surface, ligatures that secure arch wires to bracket slots are prone to microbial adhesion (Forsberg et al., 1991; Gastel et al., 2009; Papaioannou et al., 2007). Due to their advantageous handling, elastomeric polyurethane‐based ligatures are widely used by clinicians nowadays, although they are associated with higher levels of bacterial adhesion than conventional steel ligatures (Forsberg et al., 1991). Some attempts to modify elastomeric ligatures to reduce bacterial adhesion have been reported in the literature but were not found to be effective: the assessment of fluoride‐releasing elastomeric ligatures showed no long‐term clinical efficacy in reducing *S. mutans* counts in saliva or plaque (Miura et al., 2007; Wilson & Gregory, 1995). Hydrogel‐polymer coating of elastomeric ligatures to smoothen surfaces did not exhibit antibacterial properties (Magno et al., 2008).

Various dental materials including titanium implants, resin composites, and temporary filling materials have been successfully equipped with antimicrobial properties by incorporating metallic agents, with silver and bismuth particles attracting particular attention (Chen et al., 2010; Gosau et al., 2015; Hotta et al., 1998; Yamamoto et al., 1996; Yoshida et al., 1999). However, for silverized elastomeric ligatures, evidence about antimicrobial properties in vivo is conflicting. A product that releases silver ions from silver‐zeolite integrated into an elastomeric structure showed a reduction of periodontal pathogens and gingival inflammation in a study by Caccianiga et al. (2012), while no significant antimicrobial effect was reported by Kim et al. (2012). The authors suggested that the concentration of silver ions at the surface of the ligatures was insufficient for significant antimicrobial effects.

Poor surface concentrations of metallic agents could be overcome by coating material surfaces with metallic nanoparticles, which are insoluble particles with a size of less than 100 nm that show a high surface‐to‐volume ratio. This allows them to interact closely with microbial membranes and provides a large surface area for antimicrobial activity (Cushing et al., 2004; Morones et al., 2005; Verran et al., 2007). The antibacterial effect of metallic nanoparticles consists of several mechanisms including the disruption of bacterial metabolic processes, interactions with bacterial DNA, and the increase of the cytoplasmatic membrane permeability (Eckhardt et al., 2013; Feng et al., 2000; Lok et al., 2006; Morones et al., 2005).

In a recent in vitro study, elastomeric ligatures decorated with plant‐extracted silver nanoparticles showed antibacterial potential (Cabral‐Romero et al., 2013). While this elaborated technology is a sustainable approach for the future of surface treatment, easy‐to‐perform and well‐established technologies remain relevant at the present. Among those, magnetron sputtering is a widely performed state‐of‐the‐art technology used to coat textiles and biomedical products with metallic nanofilms (Alvarez et al., 2019; Berumen et al., 2019; Miśkiewicz et al., 2019; Rtimi et al., 2017; Tan et al., 2018). It combines the advantages of controllable film thickness, easy procedure, satisfactory adhesion to the target substratum, and high purity of the deposited metal (Kylián et al., 2019; Tan et al., 2018). Hence, magnetron sputtering could be an effective method to equip surfaces of elastomeric ligatures with silver and bismuth nanofilms that, to our knowledge, has not yet been assessed.

In the present in vitro study, the feasibility of coating elastomeric ligatures with silver and bismuth nanofilms by magnetron sputtering was evaluated. In addition to the surface characteristics, the antibacterial properties of the nanofilms were investigated by assessing *S. mutans* adhesion.

## 2 METHODS

### 2.1 Specimen preparation

Cylindrical specimens (diameter: 10 mm, height: 1.5 mm) were obtained from a polyurethane‐based material used for elastomeric ligatures (Sani‐Tie®, Dentsply Sirona). Specimens were cleaned with 99% isopropanol and deionized water in a 1:1 ratio with ultrasound assistance and then dried. Coating of the specimens with nanoparticles of silver and bismuth was performed by direct current magnetron sputtering (Wasa et al., 2012) at the Fraunhofer Institute for Interfacial Engineering and Biotechnology IGB using the following setup: Power supply was provided by a direct current generator (PFG 2500 DC, Trumpf Hüttinger GmbH & Co. KG). Before the metallic coating was performed, plasma activation of specimen surfaces was carried out for 1 min using radio frequency plasma (13.67 MHz) at a radio frequency power of 40 W provided by an AGC‐5 generator (Eni Power Systems) under impedance matching (viking impedance matching network, Nye Company). The subsequent coating process was carried out with a continuous flow of argon gas, which was controlled by a needle valve. The base pressure and the pressure during the coating process were continuously controlled by a ionization vacuum gauge (Ionivac Granville‐Phillips®, MKS Instruments), a vacuum gauge (Convectron Granville‐Phillips® Pirani, MKS Instruments), and a capacitance manometer (Baratron®, MKS Instruments). After reaching the base pressure of 2.1–6 mbar, argon was fed into the sputtering system and the process pressure of 6 µbar was set via the gas flow. The coating process with silver and bismuth nanoparticles was each carried out at a current of 0.4 A for 5 min. Both plasma activation and the coating process were performed under a continuous vacuum environment.

### 2.2 Surface roughness and topography

The arithmetical mean roughness values (*R*_a_) were calculated by five independent measurements using widefield confocal microscopy (Zeiss Smartproof 5, Carl Zeiss) and automated software analysis (ConfoMap ST 7.4.8076, Carl Zeiss). Surface imaging was performed by a Zeiss Object Lens C Epiplan‐APOCHROMAT (Carl Zeiss Microscopy GmbH) with 20‐fold magnification. Images of true‐color surface topography and three‐dimensional surface texture were generated with the help of ConfoMap‐Software (Carl Zeiss Microscopy GmbH).

### 2.3 Surface‐free energy

To determine surface‐free energy, at least nine independent contact angle measurements were performed: 1 µL of distilled water and 1 µL of methylene iodide were applied to the specimen's surface. Within 30 s after application, a computer‐aided measurement device (Drop Shape Analyzer DSA 25, Krüss) performed 10 contact angle measurements for each liquid. The surface‐free energy was calculated using the method by Owens and Wendt (1969).

### 2.4 Microbial culture

*S. mutans* (strain 20523; DSMZ) was cultured in sterile trypticase soy broth (Tryptic Soy Broth; BD Diagnostics) supplemented with yeast extract (Sigma‐Aldrich) at 37°C and 55 rpm for 16 h. Bacteria were harvested by centrifugation, washed twice with phosphate‐buffered saline (PBS, Merck), and resuspended in PBS. Using densitometry (Bio Photometer, Eppendorf), the suspension was adjusted to an optical density of 0.3 at 600 nm.

### 2.5 Luminescence assay

Under sterile conditions, uncoated and coated elastomeric specimens (*n* = min. 23) were transferred to 24‐well plates and attached to well bottoms using silicone (Z‐Dupe, Henry Schein Dental). Then, 1 mL of *S. mutans* suspension was added to each well and incubated for 2 h at 37°C and 55 rpm. The viable cells were quantified using an adenosine triphosphate (ATP)‐based luminescence assay (LT07‐221, Lonza): after washing with PBS twice to remove non‐adherent cells, 300 µL of a cell lysis reagent were added to each well to extract ATP. After 10 min, 100 µL of the supernatants were transferred to a 96‐well plate, where 100 µL of ATP monitoring reagent plus were added to each well. After 5 min of incubation, luminescence was measured using a plate reader (FLUOstar Omega, BMG Labtech) at a preset gain of 4000. The relative luminescence of PBS and pure bacterial solution served as control references.

### 2.6 Bacterial staining

Exemplary Hoechst staining was performed for each test group after bacterial incubation: silver and bismuth‐coated elastomeric specimens and uncoated controls were washed three times with 0.85% saline. Then, 1 mL bisbenzimide H 33342 trihydrochloride (Sigma Aldrich) was added to each specimen for 15 min. Staining solution was removed by washing three times with 0.85% saline. *S. mutans* cultures were fixated using 8% paraformaldehyde solution. Specimens were dried for 10 min, then mounted on object slides. Visualization was carried out using fluorescence microscopy (BZ‐X710, Keyence).

### 2.7 Statistical analysis

Statistical analyses were performed using GraphPad Prism 9 (GraphPad Software). The overall level for significance was set at *α* = .05. Surface roughness and surface‐free energy data are shown as means and standard deviations. Data from the ATP luminescence assay are shown as medians with box‐and‐whisker plots. For the analysis of surface roughness, surface‐free energy, and bacterial adhesion, data were tested for normal distribution (Q–Q plotting and Shapiro–Wilk test) and variance homogeneity (Levene's test). Then, one‐way analysis of variance and Tukey's multiple comparison post hoc analysis were performed. Pearson correlation analysis was used to determine the correlation between surface roughness and microbial adhesion or surface‐free energy and microbial adhesion, respectively.

## 3 RESULTS

### 3.1 Surface characteristics

**Figure 1:**
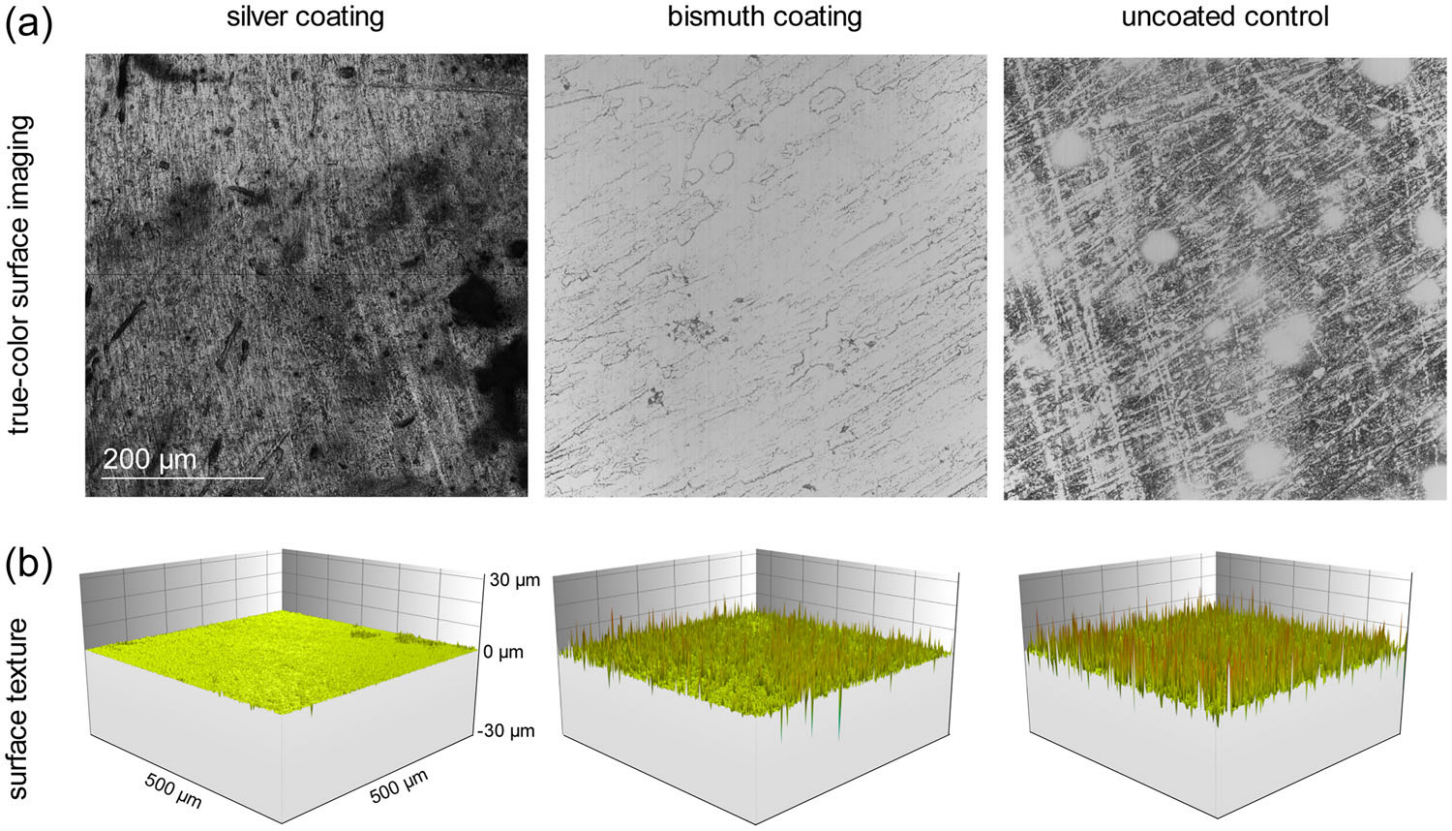
Surface characteristics of elastomeric ligatures after magnetron sputtering with silver and bismuth. (a) True‐color surface imaging shows the silver and bismuth nanofilms of the specimens. Uncoated controls show ubiquitous scratches and dents, silver coating is finer in structure with visible scratches and pits, bismuth coating displays the most homogeneous surface. (b) Three‐dimensional surface texture reveals smoother surfaces of silver‐coated specimens compared to bismuth‐coated and uncoated specimens indicated by peaks with small amplitudes.

Exemplary true‐color surface imaging showed the surface topography of the elastomeric ligatures after deposition of silver and bismuth nanofilms by magnetron sputtering (Figure 1a). The uncoated controls showed ubiquitous scratches and dents. Silver‐coated surfaces were more finely textured with visible pits and scratches while bismuth coating displayed more homogeneous surfaces. Three‐dimensional surface texture imaging revealed smoother surfaces of silver and bismuth‐coated elastomeric ligatures compared to uncoated controls (Figure 1b). Corresponding arithmetic mean roughness (*R*_a_) and surface‐free energy are shown in Table 1. Silver sputtered surfaces were significantly smoother (*p* < .0001) than uncoated and bismuth coated ligatures. Bismuth sputtered surfaces were not significantly smoother than uncoated controls (*p* = .328). Both silver and bismuth sputtered surfaces showed significantly higher surface‐free energy values than uncoated controls (*p* < .0001). Sputtering ligatures with bismuth significantly increased surface‐free energy compared to sputtering with silver (*p* < .001).

**Table 1:** Surface characteristics of the investigated elastomeric ligature modifications.

Surface modification	*R*_a_ (µm)	Surface free energy (mN/m)
Silver coating	0.31 ± 0.01^ab^	69.69 ± 1.41^cd^
Bismuth coating	2.15 ± 0.11^a^	73.92 ± 1.48^ce^
Uncoated control	2.05 ± 0.15^b^	62.93 ± 3.78^de^

*Note*: Data are expressed as means and standard deviations. Equal letters indicate significant differences (see text for level of significance).

### 3.2 Bacterial adhesion

**Figure 2:**
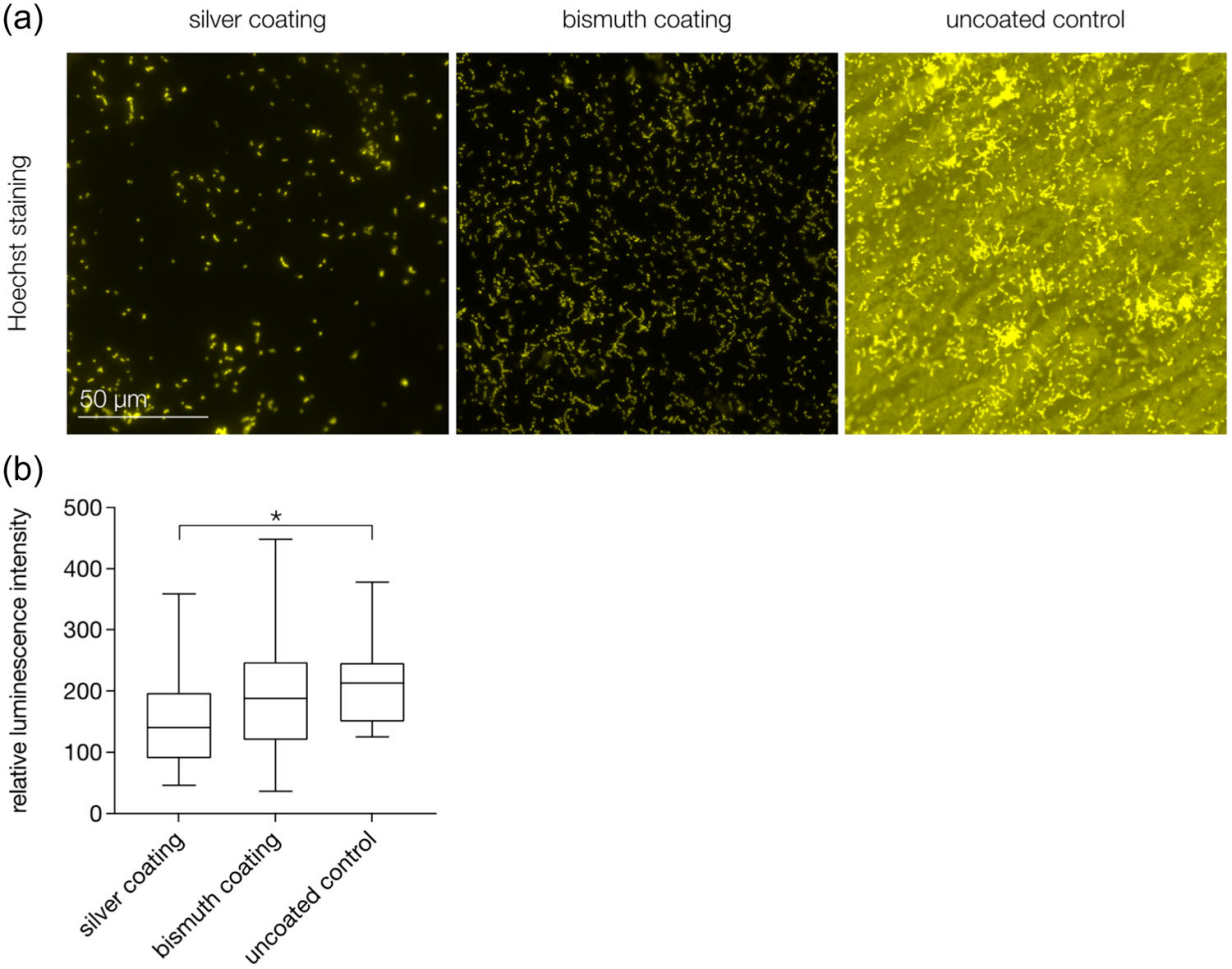
Adhesion of *Streptococcus mutans* to the investigated silver‐ and bismuth‐coated elastomeric specimens. (a) Hoechst staining shows typical chain formations of *S. mutans* cells. The surface of the uncoated control shows strong auto fluorescence. There is a tendency for lower bacterial accumulation on silver‐coated specimens. (b) Results of the luminescence assay show significantly lower adhesion of *S. mutans* to silver‐coated specimens compared to uncoated controls. *p < .05.

Hoechst staining (Figure 2a) was performed to visualize the adhesion of *S. mutans* to the investigated coated and uncoated elastomeric ligatures. *S. mutans* cells accumulated in typical chain formations on the surfaces of the specimens. There was a tendency for lower bacterial adhesion on silver‐coated specimens. The ATP assay (Figure 2b) revealed significantly less bacterial adhesion to silver‐coated specimens than to control specimens. Bismuth coating did not result in significantly reduced adhesion of *S. mutans*.

Pearson correlation analysis showed a positive but insignificant correlation between surface roughness and bacterial adhesion (correlation coefficient *r* = .9821, *p* = .1205). There was no correlation between surface‐free energy and bacterial adhesion (*r* = −.2688, *p* = .8267).

## 4 CONCLUSION

Fixed orthodontic devices tend to accumulate microorganisms and complicate patients’ oral hygiene. White spots and carious lesions are undesirable biofilm‐associated side effects of fixed orthodontic therapy (Bergstrand, 2011; Lovrov et al., 2007; Ogaard et al., 1988; O'Reilly & Featherstone, 1987; Richter et al., 2011). To equip the surfaces of orthodontic elastomeric ligatures with antimicrobial properties, the present study assessed magnetron sputtering as a method to deposit silver and bismuth nanofilms on orthodontic elastomeric ligatures.

Widefield confocal microscopy revealed successful deposition of nanofilms on the surface of elastomeric ligatures. Surface imaging showed homogeneous surfaces in bismuth‐coated specimens. Silver coating was heterogeneous but finer in texture than uncoated controls which showed ubiquitous scratches and dents. In contrast to the visual impression, surface roughness was significantly decreased by silver‐sputtering while bismuth‐sputtering had no significant effect on surface roughness compared to uncoated controls. This finding is in line with a study on titanium implants by Gosau et al., in which bismuth coating by magnetron sputtering was associated with rougher surfaces than silver coating, possibly due to the crystal nanostructure of bismuth (Gosau et al., 2015) that was not detectable by confocal microscopy in the present study.

Adhesion of *S. mutans* to coated and uncoated specimens was visualized by Hoechst staining, an established method for assessing bacterial colonization (Dai et al., 2020; Schubert et al., 2021; Yang et al., 2021). A state‐of‐the‐art ATP‐based luminescence assay with high reproducibility and sensitivity was performed to quantify the adhered bacteria (Almohandes et al., 2021; Crouch et al., 1993; Dexter et al., 2003; Hahnel et al., 2008; Schubert et al., 2021; Wassmann et al., 2020). Silver coating resulted in a significant decrease of *S. mutans* adhesion compared to uncoated controls. Similar antimicrobial effects of silver have been reported for various dental materials. Resin composites loaded with high concentrations of silver‐containing fillers showed significant inhibitory effects on *S. mutans* growth (Yoshida et al., 1999). Titanium implants coated with silver showed antimicrobial effects against *S. epidermidis* (Gosau et al., 2015) and silver‐containing temporary filling materials exhibited antibacterial activity against oral *streptococci* in vitro (Hotta et al., 1998; Yamamoto et al., 1996). In elastomeric ligatures, however, the antimicrobial effect of silver ions within a porous crystalline network was insignificant against *S. mutans* (Kim et al., 2012). Low surface concentrations of silver ions have been discussed as a possible reason for the insufficient antimicrobial effects. From the results of the ATP‐assay performed in the present study, it can be assumed that magnetron sputtering is a suitable method to equip surfaces of elastomeric ligatures with sufficiently high concentrations of silver nanoparticles to achieve antimicrobial effects.

Bismuth coating showed a tendency for reduced adhesion of *S. mutans* compared to uncoated controls. However, the effect was weaker than the one exerted by silver. Due to a lack of studies, our results cannot be compared with other findings on bismuth‐coated orthodontic devices. However, there are multiple investigations on bismuth‐coated dental implants with both significant and insignificant antibacterial effects being reported (Almohandes et al., 2021; Ciobanu & Harja, 2019; Gosau et al., 2015; Lin et al., 2013). In calcium phosphate cement, bismuth had a potent antimicrobial activity relevant for root canal fillings. The antibacterial effect of bismuth remains controversial and needs further investigation. Based on our data, silver coating is preferable to bismuth coating to achieve antibacterial effects in elastomeric ligatures.

For the interpretation of the results from the adhesion assay, the influence of surface properties on microbial adhesion should be considered in addition to the effects attributed to the metallic nanoparticles. The influence of surface roughness on microbial adhesion to dental materials has been widely discussed in the literature, and, in general, rough surfaces are more susceptible to bacterial adhesion than smooth surfaces (Quirynen et al., 1993, 1996; Teughels et al., 2006; Yoda et al., 2014). This can be explained by larger surface areas exhibited by rough surfaces which increases the absolute number of bacterial cells and by better protection of bacteria from shear forces which facilitates initial cell adhesion (Scheuerman et al., 1998; Teughels et al., 2006). Reducing surface roughness of elastomeric ligatures to minimize bacterial adhesion has already been subject of research: Magno et al. performed an in vivo study on elastomeric ligatures coated with a hydrogel‐polymer that is claimed to turn into a highly smooth surface when moistened. However, adhesion of *S. mutans* was not reduced by this surface modification, possibly due to detachment of the coating and fissures in the surface after traction of ligatures during clinical application (Magno et al., 2008). In contrast to this finding, Pearson correlation analysis in the present study showed a positive, albeit not significant, correlation between surface roughness and bacterial adhesion. This tendency suggests that the significantly lower adhesion of *S. mutans* to silver‐coated ligatures compared with uncoated controls may be explained in part by the significantly lower surface roughness.

In addition to surface roughness, surface‐free energy is a predominant factor influencing microbial adhesion to solid surfaces and has been assessed for various dental materials (Arima & Iwata, 2007; Hahnel et al., 2008; Lee et al., 1998). Depending on the experimental settings and conditions, variations of surface‐free energy may have stimulating or reducing effects on microbial adhesion (D'Ercole et al., 2020; Wassmann et al., 2017; Zhao et al., 2004). For the adhesion of *S. mutans* to dental composite resins, both insignificant and significant correlations with surface‐free energy have been shown (Bilgili et al., 2020; Jeon et al., 2020; Mandracci et al., 2015). To our knowledge, there is currently no evidence about the effect of surface‐free energy on the adhesion of *S. mutans* to elastomeric ligatures. In the present study, both silver and bismuth coating resulted in a significant increase of surface‐free energy. Yet, statistical analysis suggests no significant correlation between surface‐free energy and the adhesion of *S. mutans* in the performed investigations. The present data on silver and bismuth‐coated elastomeric ligatures indicate that surface roughness had a greater influence on bacterial adhesion than surface‐free energy. Details about the complex interaction of biological and physicochemical mechanisms behind microbial adhesion to dental materials, especially those coated with metallic nanofilms, remain to be elucidated by future research.

As mentioned, caries can be seen as a potential consequence of a dysbiosis of the oral microbiome (Atack et al., 1996; Johansson et al., 2016). This implies a process of complex interaction of the oral microbiota, which can be illustrated by the successive maturation and growth phases of an oral biofilm. A mature multispecies biofilm exhibits properties, for example, resistance to environmental changes, that individual species do not (Kolenbrander et al., 2010; Wang et al., 2020). The interactions in a biofilm, which can ultimately become cariogenic, are therefore the subject of current research and are difficult to simulate under in vitro conditions. The exact identification of the cariogenic microorganisms is also inconclusive, but Streptococcus mutans, as examined in this study, has the highest detection rate in caries patients (Bhaumik et al., 2021; Ev et al., 2023). An exemplary observation of a single species therefore does not represent the complexity or resilience of a biofilm, but can provide reliable findings before more complex or even in vivo tests.

Generally, the present study showed the feasibility of the investigated magnetron sputtering procedure. Silver and bismuth nanofilms were successfully deposited onto the surface of elastomeric ligatures. However, the nature of both metallic coatings was rather unstable as stretching or moistening of the coated ligatures resulted in partial detachment of the nanofilms, a phenomenon that has been described for hydrogel‐polymer coatings as well (Magno et al., 2008). Under in vivo conditions, ligatures are stretched to place them around brackets. Moreover, they are exposed to stresses like chewing, tonicity of the lips, saliva, and oral hygiene procedures. Based on the results of the present investigation, it can be assumed that the adhesion of the silver and bismuth nanoparticles to the ligature surface is not sufficient to withstand the clinical conditions during orthodontic treatment. Hence, the conclusions drawn from the present in vitro study must be considered preliminary until in vivo data is available. Considering the cytotoxic potential of metallic nanoparticles, their detachment and swallowing may cause adverse local or systemic side effects (Fu et al., 2014; Pérez‐Díaz et al., 2015; Składanowski et al., 2016; Zhang et al., 2014). While the present study showed the potential to reduce microbial adhesion to elastomeric ligatures by silver and bismuth nanofilms, future research is necessary to provide stable and lasting coatings. Also, their biocompatibility should be assessed by evaluating cytotoxic effects.

The present in vitro study suggests that magnetron sputtering is a feasible method to deposit silver and bismuth nanofilms to orthodontic elastomeric ligatures. Silver nanofilms exerted a significant antibacterial effect with potential clinical relevance to reduce white spots and carious lesions in orthodontic patients. Future research is required to stabilize the metallic coating to withstand in vivo conditions.

## AUTHOR CONTRIBUTIONS

**Andrea Schubert**: Validation; formal analysis; writing—original draft; visualization. Carolin Griesmüller: Formal analysis; investigation. **Nikolaus Gersdorff**: Conceptualization. **Ralf Bürgers**: Conceptualization; methodology; resources; writing—review and editing; supervision; project administration. **Bernhard Wiechens**: Investigation; writing—review and editing; supervision. **Torsten Wassmann**: Methodology; validation; formal analysis; writing—review and editing; visualization; project administration. All authors have read and agreed to the published version of the manuscript.

## CONFLICT OF INTEREST STATEMENT

The authors declare no conflict of interest.

## Data Availability

The data presented in this publication are openly available in the data repository “Göttingen Research Online” (https://doi.org/10.25625/DOSOZ9).
